# Unknown components of the plastidial permeome

**DOI:** 10.3389/fpls.2014.00410

**Published:** 2014-08-19

**Authors:** Thea R. Pick, Andreas P. M. Weber

**Affiliations:** Institut für Biochemie der Pflanzen, Cluster of Excellence on Plant Sciences, Heinrich-Heine Universität DüsseldorfDüsseldorf, Germany

**Keywords:** chloroplast envelope membrane, metabolite transport, permeome

## Abstract

Beyond their role in photosynthesis plastids provide a plethora of additional metabolic functions to plant cells. For example, they harbor complete biosynthetic pathways for the *de novo* synthesis of carotenoids, fatty acids, and amino acids. Furthermore plastids contribute important reactions to multi-compartmentalized pathways, such as photorespiration or plant hormone syntheses, and they depend on the import of essential molecules that they cannot synthesize themselves, such as ascorbic acid. This causes a high traffic of metabolites across the plastid envelope. Although it was recently shown that non-polar substrates could be exchanged between the plastid and the ER without involving transporters, various essential transport processes are mediated by highly selective but still unknown metabolite transporters. This review focuses on selected components of the plastidial permeome that are predicted to exist but that have not yet been identified as molecular entities, such as the transporters for isopentenyl diphosphate (IPP) or ascorbic acid.

## INTRODUCTION

Both, plastids and mitochondria are of endosymbiotic origin and play a pivotal role in energy supply and metabolism of plant cells. Both organelles harbor a plethora of metabolic processes and exchange a high amount of metabolites with the surrounding cell. Plastids are the site of photosynthesis, fatty acid synthesis, amino acid synthesis, isoprenoid synthesis, as well as sulfur and nitrogen assimilation. In addition, they contribute to pathways that are distributed across several compartments, such as photorespiration, pyrimidine synthesis, and hormone syntheses, leading to a high flux of metabolites over the plastid envelope.

The envelope of plastids of primary endosymbiotic origin consists of two distinct membranes, namely the outer (OE) and the inner envelope (IE) membranes. While the IE was understood as the *de facto* permeability barrier between cytosol and plastid, the OE was regarded as an unspecific membrane permeable for molecules smaller than 10 kDa. By now it is known that the OE is a metabolically active part of plastids (Schnurr et al., 2002) and contains pore forming proteins of different selectivity, like OEP16 (for outer envelope protein of 16 kDa; Pohlmeyer et al., 1997; Pudelski et al., 2012), OEP21 (Bolter et al., 1999), OEP24 (Pohlmeyer et al., 1998), and OEP37 (Goetze et al., 2006). Still it is controversial if the OE actively participates in the selective transport of metabolites (Flugge, 2000; Bolter and Soll, 2001).

Because a controlled exchange of metabolites across organellar boundaries is a pivotal requisite for proper function of plant metabolism the activity of specific transport proteins is indispensable. A prominent example for plastid envelope transporters is the triose phosphate/phosphate translocator (TPT; Flugge et al., 1989). The TPT belongs to the family of plastidic phosphate translocators and was the first transporter of the plastid envelope membrane that was identified at the molecular level and biochemically characterized in a heterologous system.

Recently it was shown that exchange of non-polar substrates between the plastid and the ER can proceed without the involvement of specific transport proteins by a membrane hemi-fusion mechanism (Mehrshahi et al., 2013). However, more than 100 putative plastidial transporters have been identified by *in silico* methods (Weber et al., 2005; Armbruster et al., 2011) and various chloroplast inner and outer envelope proteins that were detected by proteomics or transcriptomics are still awaiting functional characterization (Brautigam et al., 2008; Brautigam and Weber, 2009; Manandhar-Shrestha et al., 2013).

Hence, assuming that the abovementioned estimates are sensible, approximately 75% of the plastidial translocators remain unidentified as molecular entities. An example for the obstacles on the way to success is the recently identified photorespiratory glycolate glycerate transporter (Pick et al., 2013). The existence of this transport protein and its transport mode were established by biochemical assays already in the 1980s (Howitz and McCarty, 1985a,b, 1986). Nevertheless it took several decades to eventually link the biochemical function to a molecular entity, which became possible through multivariate statistical analyses of *Arabidopsis* gene expression datasets (Pick et al., 2013).

**FIGURE 1:**
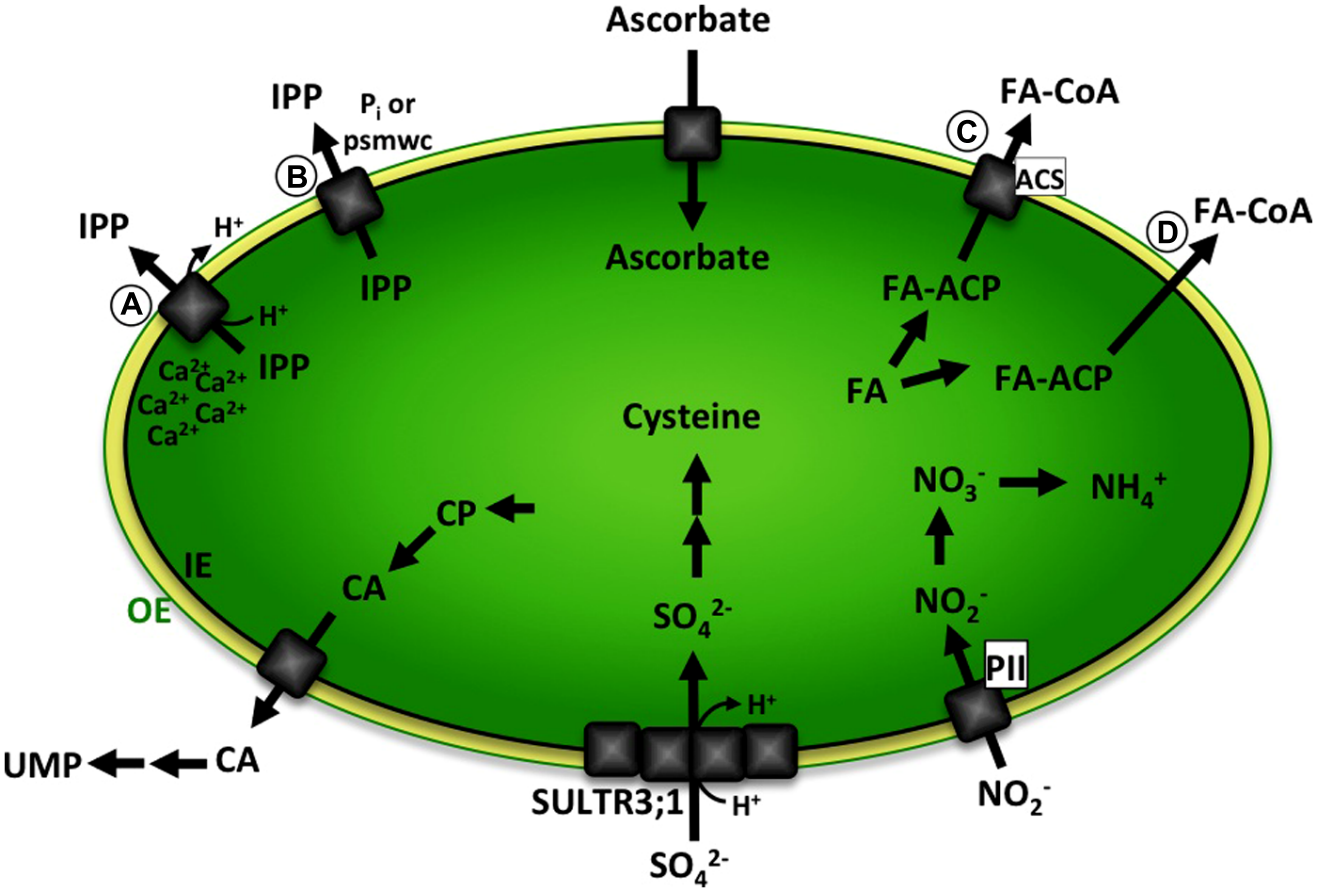
**Schematic representation of biochemical pathways with predicted metabolite transport steps over the plastid envelope discussed in this review.** Ascorbate is exclusively produced in mitochondria and transported into plastids. IPP is either **(A)** exported in a Ca^2+^-gated IPP/proton symport mechanism or **(B)** transport absolutely depends on binding of phosphorylated small molecular weight compounds (psmwc) to a regulatory side on the *trans* side of the membrane. Nitrite transport likely involves binding of plastidic PII protein. Sulfate is imported by a sulfate/proton cotransporter and is partially mediated by SULTR3;1, presumably involving other members of the SULTR3 family or a second independent transport system. CA is produced from CP in the plastid and exported to the cytosol. Further enzymatic steps in the cytosol and mitochondria lead to the formation of UMP. Fatty acids are synthesized in the plastid, attached to a ACP and exported either **(C)** involving a transport protein and binding of ACS in the outer envelope or **(D)** by facilitated diffusion leading to formation of FA–CoA. The green line represents the plastid outer envelope membrane (OE). The black line represents the plastid inner envelope membrane (IE). Black arrows indicate enzymatic steps. Gray squares represent transport proteins. OE, plastid outer envelope membrane; IE, plastid inner envelope membrane; psmwc, phosphorylated small molecular weight compounds; IPP, Isopentenyl diphosphate; PII, plastidic PII protein; CP, Carbamoyl phosphate; CA, Carbamoyl aspartate; UMP, uridine monophosphate; FA, free fatty acids; FA-ACP, fatty acid – Acyl carrier protein; ACS, acyl-coenzyme A (CoA) synthetase.

In this review we will focus on a subset of those unknown transporters, hence summarizing a selection of biochemical pathways with predicted metabolite transport steps over the plastid envelope (**Figure 1**).

## TRANSPORT OF ASCORBIC ACID

In plants, ascorbate is present in all tissues and cell compartments (Zechmann et al., 2011). Up to four routes lead to the formation of ascorbate, including the L-galactose-pathway, the animal-like pathway via *myo*-inositol/glucuronate, the salvage pathway via L-galactonate, and the L-gulose-pathway. Among these four routes the biosynthetic route via L-galactose, also called the Smirnoff–Wheeler pathway, is the major pathway in many plants (Wheeler et al., 1998; Gatzek et al., 2002; Laing et al., 2007; Bulley et al., 2009). In this pathway D-glucose is converted via the intermediates GDP-D-mannose and L-galactose to L-ascorbate. Only recently the last missing enzyme in this pathway was identified, namely VTC2, a GDP-L-galactose phosphorylase (Laing et al., 2007). VTC2 was shown to be the rate-limiting step in ascorbate biosynthesis and overexpression of the VTC2 orthologous from kiwifruit (*Actinidia* spp.) in *Arabidopsis* resulted in an up to fourfold increase in ascorbate levels (Bulley et al., 2009). In addition, transient overexpression of VTC2 in *Arabidopsis* revealed its function as a major control point in light/dark regulation of ascorbate biosynthesis (Yoshimura et al., 2014). Except the last enzymatic step of ascorbate biosynthesis, the conversion of L-galactono-1,4-lactone to L-ascorbate by L-galactono-1,4-lactone dehydrogenase (GLDH) on the inner mitochondrial membrane all enzymatic reactions take place in the cytosol. Among the other pathways the salvage pathway via L-galactonate was recently shown to essentially contribute to acsorbate levels in ripening tomato fruits (Badejo et al., 2012).

Since the last step of all four routes to ascorbate synthesis occurs on the inner mitochondrial membrane, transport into other organelles is essential (Horemans et al., 2000; Smirnoff and Wheeler, 2000). Moreover, ascorbate was shown to be distribute throughout the plant via the phloem from source to sink tissues (Franceschi and Tarlyn, 2002). Ascorbate is involved in many pathways, such as hormone biosynthesis, regenerating of other antioxidants, cell division and growth, and signal transduction. Furthermore, a recent study in pea and *Arabidopsis* embryos showed that ascorbate is crucial for iron uptake, as ascorbate-mediated reduction is an obligatory step for the uptake of iron(II; Grillet et al., 2014). Ascorbate is essential for proper chloroplast function, since it acts as redox buffer in the Mehler peroxidase reaction with ascorbate peroxidase (APX) and thus regulates photosynthesis (Neubauer and Yamamoto, 1994; Caverzan et al., 2014). Hence, in photosynthetically active chloroplasts ascorbate concentration can reach up to 20 mM (Foyer and Lelandais, 1996; Zechmann et al., 2011), which requires a high flux of this metabolite across the plastid envelope.

In plants, the best-characterized ascorbate transporter resides in the plasma membrane. It was characterized in isolated plasma membrane vesicles of *Phaseolus vulgaris L.*, indicating an ascorbate/dehydroascorbate antiport mechanism (Horemans et al., 1996). Transport over the plastid envelope was determined biochemically. Uptake assays using intact isolated chloroplasts from *Spinacia oleracea* and *Pisum sativum* revealed that ascorbate is taken up by a saturable carrier that has a very low affinity (*K*_m_ = 20 mM), and does not transport glucose (Beck et al., 1983; Foyer and Lelandais, 1996).

Recently Maurino et al. (2006) identified the nucleobase-ascorbate transporter (NAT) gene family in *Arabidopsis thaliana* and rice (*Oryza sativa*) and Cai et al. (2014) in tomato (*Solanum lycopersicum*). Members of the NAT family share similarities with both transporters involved in uptake of nucleobases from fungi and the well characterized mammalian sodium-dependent ascorbate transporters (Gournas et al., 2008; Buerzle et al., 2013), making the members of the plant NAT family promising candidates for ascorbate transporters. Interestingly, AtNAT12 along with OsNAT10 and OsNAT11 were predicted to be plastid localized but subcellular localization of AtNAT12 indicate that it is localized to the plasma membrane (Maurino et al., 2006). Yet, ascorbate transport activity of plant NAT family members still remains to be demonstrated and the molecular nature of the plastid ascorbate transporter remains elusive.

## ISOPENTENYL DIPHOSPHATE (IPP)

Isoprenoids are essential metabolites found in all organisms. In plants, isoprenoid-derived compounds such as carotenoids, tocopheroles, sterols, and terpenes fulfill numerous biochemical functions in pivotal processes, including photosynthesis, membrane and hormone synthesis, and plant defense. While the universal five-carbon precursor IPP is produced via the mevalonic acid (MVA) pathway in animals and fungi and the methylerythritol phosphate (MEP) pathway in many bacteria, both pathways occur, albeit in different compartments, in plants (Lange et al., 2000). Here, IPP synthesis from acetyl-CoA via the MEP pathway operates in the cytosol (McGarvey and Croteau, 1995), whereas IPP and its double bond isomer dimethylallyl diphosphate (DMAPP) are synthesized from triose phosphates and pyruvate in plastids via the 1-deoxy-D-xylulose 5-phosphate/methylerythritol phosphate (DOXP/MEP) pathway (Lichtenthaler et al., 1997). There is experimental evidence that both pathways cooperate in synthesis of specific metabolites (Adam et al., 1999). By inhibiting either the MEP or the MVA pathway, a metabolic crosstalk between both distinct pathways was shown, suggesting transporter mediated exchange of IPP between plastids and the cytosol (Laule et al., 2003). Also, a reduction of the import of the MEP pathway precursor pyruvate into chloroplasts in *Arabidopsis* mutant lines lacking the activity of the plastidial pyruvate:sodium symporter only led to a phenotype when the cytosolic MVA pathway was repressed in the mutant lines by application of the inhibitor mevastatin (Furumoto et al., 2011). However, the mode of IPP transport still remains controversial. Bick and Lange (2003) used intact chloroplasts and envelope membrane vesicles from *Spinacia oleracea* to identify the transport mode. They proposed a Ca^2+^-gated IPP/proton symport mechanism. On the other hand, Flugge and Gao (2005) showed that IPP transport over the plastid envelope proceeds via an uniport rather than an antiport mechanism and transport absolutely depends on binding of phosphorylated compounds to a regulatory side on the *trans* side of the membrane. However, to date none of the tested plastid envelope transporters mediates IPP transport, pointing to the existence of a yet unknown transporter for IPP.

## NITRITE AND SULFUR

In plants primary ammonium (${NH}_{4}^{+}$) and sulfate assimilation takes place in chloroplasts. Nitrate (${NO}_{3}^{-}$) is taken up and reduced to nitrite (${NO}_{2}^{-}$) in the cytosol of mesophyll cells where nitrite is eventually taken up into chloroplast and further reduced to ammonium (Stitt et al., 2002). Uptake studies with chloroplast envelope membrane vesicles first pointed to rapid diffusion of nitrite, in the form of nitrous acid, across membranes without involvement of a transport protein (Shingles et al., 1996). However, uptake studies with intact chloroplasts revealed saturation kinetics that were highly comparable with those observed for nitrite reduction in chloroplasts, indicating a transporter mediated and regulated uptake of nitrite (Brunswick and Cresswell, 1988a,b). A nitrite transporter candidate, Nitr1-L, was shown to be chloroplast localized and *Arabidopsis* knockout mutants exhibited a five times higher accumulation of nitrite in leaves compared to wild type plants, pointing to a nitrite transport function of Nitr1-L (Sugiura et al., 2007). Moreover, it was shown that the plastidic PII protein is involved in nitrite uptake through a yet unknown mechanism (Ferrario-Mery et al., 2008). However, interaction of PII with Nitr1-L and biochemically direct nitrite uptake by Nitr1-L still remains to be demonstrated. In addition, a recent study localized Nitr1-L to the plasma membrane in grapevine and *Arabidopsis*, which makes it unlikely that this protein represents the plastidial nitrite transporter (Pike et al., 2014).

Recently AtNITR2;1, a transporter belonging to the HPP protein family, was identified as a new nitrite transporter candidate (Maeda et al., 2014). AtNITR2;1 is localized to the plastid IE (Ferro et al., 2003), expressed in shoot and root, and seems to be conserved in vascular plants (Maeda et al., 2014). When expressed in NA4, the nitrite transport-less mutant of the cyanobacterium *Synechococcus elongatus* AtNITR2;1 exhibited a high affinity for nitrite (*K*_m_ = 13 μM). Additionally, *nitr2;1* *Arabidopsis* knockout mutants showed reduced uptake of nitrite into isolated chloroplasts compared to WT levels. From these experiments it was calculated that NITR2;1 accounts for ~60% of nitrite uptake at 50 μM external nitrite. However, since *nitr2;1* mutant plants did not exhibit a visible phenotype when grown on nitrate as sole nitrogen source (Maeda et al., 2014), the physiological role of NITR2;1 still needs to be further investigated.

Sulfate is reduced and assimilated into cysteine and methionine in the chloroplast. Hence, sulfate has to be transported from the cytosol into chloroplasts. Transport studies revealed a sulfate import via a proton/sulfate co-transporter (Buchner et al., 2004). Recently a functional plastidic sulfate transporter SULTR3;1 was identified by knockout mutant analysis in *Arabidopsis* (Cao et al., 2013). SULTR3;1 belongs to the SULTR3 subfamily of plant sulfate transporters that is composed of five members (Takahashi et al., 2000). In yeast complementation studies SULTR3;1, SULTR3;2, and SULTR3;3 failed to complement the phenotype of the yeast mutant lacking both sulfur transporters (Takahashi et al., 2000), probably due to incorrect targeting of the proteins. However,*in organello* transport assays confirmed the proton/sulfate symport but revealed the existence of further plastidic sulfate transporters in addition to SULTR3;1 (Cao et al., 2013). *In silico* analysis revealed similarities in genomic organization of *Sultr3;1* and *Sultr3;2* pointing to functional redundancy of SULTR3 subfamily members (Takahashi et al., 2000; Cao et al., 2013). Absence of strong phenotypes in single mutant plants additionally point to functional redundancy (Zuber et al., 2010). However, array data point to a low expression of SULTR3;2 throughout the plant (EFP browser *Arabidopsis*, Winter et al., 2007) implying the possibility of an additional transport system independent of SULTR3 family members. Further analysis of *sultr3;1 sultr3;2* double and *sultr3;1 sultr3;2 sultr3;3 sultr3;4* quadruple mutants may reveal how these transporters contribute to plastidic sulfate import.

## PYRIMIDINES

Nucleotides, including purines and pyrimidines, are crucial components for plant primary and secondary metabolism and development. Pyrimidine *de novo* synthesis is a highly compartmentalized pathway involving enzymatic reactions in plastids, mitochondria, and the cytosol. The initial steps take place in plastids, eventually leading to the formation of carbamoyl aspartate (CA; Shibata et al., 1986; Chen and Slocum, 2008). In contrast to previous assumptions, where the conversion from CA to dihydroorotate (DHO) by DHOase took place in the plastids, protein-GFP fusion analysis could clearly localize the DHOase to the cytosol (Witz et al., 2012). This implies that CA has to be exported from plastids into the cytosol. In addition to *de novo* synthesis pyrimidines can also be synthesized via the less energy consuming salvage pathway. Presumably *de novo* synthesis in plants occurs in dividing cells and growing tissues where a high amount of nucleotides is required, whereas the salvage pathway operates in non-growing cells and mature tissues. Two studies revealed that the salvage pathway plays an essential role in the supply of pyrimidines to the plant (Mainguet et al., 2009; Chen and Thelen, 2011). Here it was shown that single mutant plants lacking plastidic uracil phosphoribosyltransferase (UPRT; Mainguet et al., 2009) and double mutant plants lacking both plastidic isoforms of uridine kinase (UKL1 and UKL2; Chen and Thelen, 2011) display dwarfish and chlorotic phenotypes.

Recently the plastidic uracil transporter PLUTO (plastidic nucleobase transporter) that is involved in the salvage pathway was identified (Witz et al., 2012). However, the transport mode of CA export during *de novo* synthesis and a transport protein remain unknown. As CA is a modified amino acid, transport through the DIT protein family, members of the plastidic amino acid permease (AAP) family, or the plant preprotein and amino acid transporter (PRAT) superfamily might be possible (Renne et al., 2003; Weber et al., 2005; Murcha et al., 2007).

## AMINO ACIDS

Amino acids are the building blocks of enzymes and proteins and fulfill various additional functions in plants. They act as nitrogen donors for a variety of essential compounds and play an indispensable role in important processes like photorespiration. Plants are able to *de novo* synthesize all 20 proteogenic amino acids, while several enzymes involved in the biosynthesis of amino acids reside in the plastids and many amino acids are produced here (Kirk and Leech, 1972; Ravanel et al., 2004). Recently it was shown that the enzymatic steps of amino acid biosynthesis in plastids predominantly consist of non-cyanobacterial enzymes (Reyes-Prieto and Moustafa, 2012). Since protein synthesis occurs in the cytosol, plastids, and mitochondria, a high flux rate of amino acids across the plastid envelope is required, at least during developmental stages that are associated with high rates of protein biosynthesis. Hence, specific import and export proteins are required. To date, the only characterized plastidic IE amino acid transporter is AtDiT2.1 (Renne et al., 2003). AtDit2.1 mediates the exchange of glutamate/malate and also accepts the amino acid aspartate as transport substrate (Werner-Washburne and Keegstra, 1983, 1985). Based on several studies, in particular proteomics, transporter candidates for plastidic amino acid transporters were identified (Kleffmann et al., 2004; Brautigam et al., 2008). For example members of the PRAT superfamily PRAT1 and PRAT2 were shown to be plastid-localized (Murcha et al., 2007) but their transport function remains to be demonstrated.

## FATTY ACIDS

In plants, plastids are the site of *de novo* fatty acid synthesis. Following synthesis, fatty acids are activated by a plastid localized acyl carrier protein (acyl-ACP) and are thus prepared for further complex lipid assembly (Ohlrogge et al., 1979). In *Arabidopsis* leaf mesophyll cells 62% of the chloroplastidic produced fatty acids are exported (Browse et al., 1986). In non-photosynthetic tissues and developing seeds of all plants even 90% of the fatty acids are exported (Browse et al., 1993). However, the molecular mechanism of fatty acid export from plastids is still controversial. It was proposed that membrane contact sites between plastids and endoplasmatic reticulum were involved (Andersson et al., 2007a,b) although experimental evidence is still missing. It has been shown, that before leaving the plastid, attaching the fatty acid to an acyl-ACP and fatty acid activation by acyl-coenzyme A (CoA) synthetase (ACS) forming fatty acyl-CoAs is mandatory (Ohlrogge et al., 2000). Both, acyl-ACP thioesterase and ACS were shown to be plastid localized with a clear localization of ACS to the plastid outer envelope (Andrews and Keegstra, 1983; Schnurr et al., 2002; Breuers et al., 2012). Recent rapid kinetic label experiments suggested that phosphatidylcholine participates in the export of newly synthesized acyl chains from plastids (Tjellstroem et al., 2012). But it is still under debate if fatty acid export is mediated by facilitated diffusion or specific transport proteins. The latter mechanism was already shown to occur in bacteria and yeast and involve activity of ACS proteins (Black and DiRusso, 2003). Recently it was also shown that the cyanobacterial ACS SLR1609 mediates the transport of fatty acids across a biological membrane (von Berlepsch et al., 2012). Transporter candidates in *Arabidopsis* were previously suggested but biochemical evidence for their involvement in fatty acid transport remains elusive (Koo and Ohlrogge, 2002).

## CONCLUSION

Over the past decade substantial effort was invested in the identification of additional plastid envelope metabolite translocators as molecular entities. These recent studies led to the molecular identification of several transporters that were previously predicted on the basis of biochemical evidence, such as the plastidial pyruvate and glycolate/glycerate transporters (Furumoto et al., 2011; Pick et al., 2013). Recent “omics” and bioinformatics approaches, such as coexpression analysis, lead to the emergence of further candidates for putative transporters in the last years that, however, still await functional characterization (Brautigam et al., 2008; Brautigam and Weber, 2009; Majeran et al., 2010; Bordych et al., 2013; Manandhar-Shrestha et al., 2013).

## Conflict of Interest Statement

The authors declare that the research was conducted in the absence of any commercial or financial relationships that could be construed as a potential conflict of interest.
